# Supplementary figures and images for: Quantitative transportomics identifies Kif5a as a major regulator of neurodegeneration
Source: eLife. 2022 Mar 8;11:e68148. doi: 10.7554/eLife.68148 (PMC8947766; doi:10.7554/eLife.68148)

Figure 1e

Raw Ponceau S

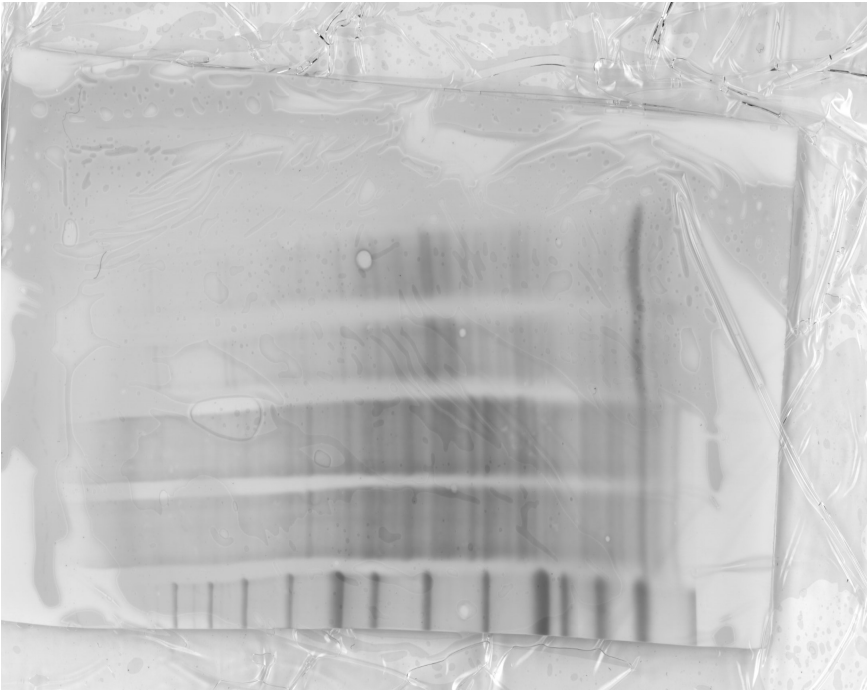

|                                                                                    | Retina |   | ON |   |
|------------------------------------------------------------------------------------|--------|---|----|---|
| ONC                                                                                | -      | + | -  | + |
| 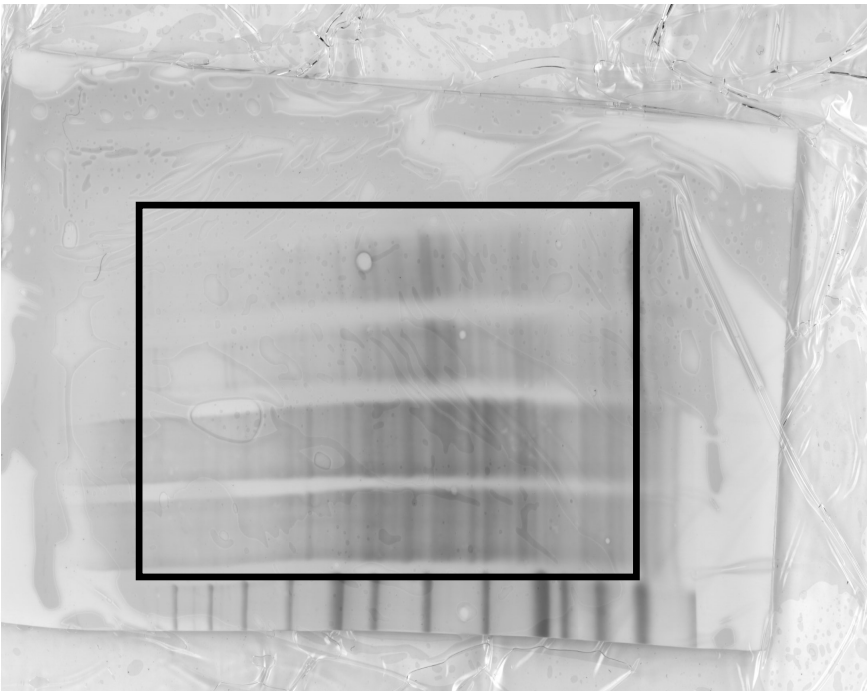 |        |   |    |   |

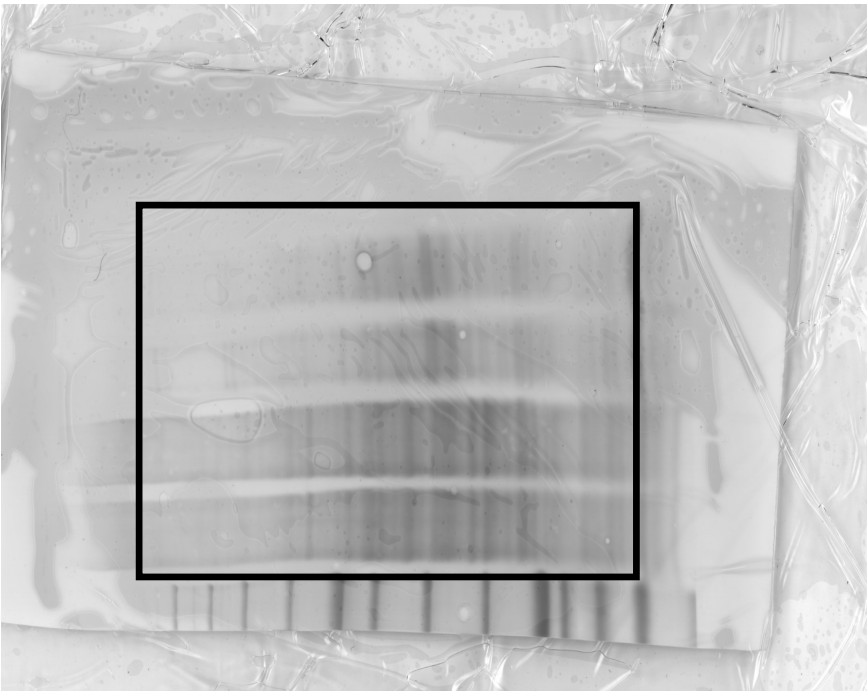

Figure 1f

Raw Western Blot

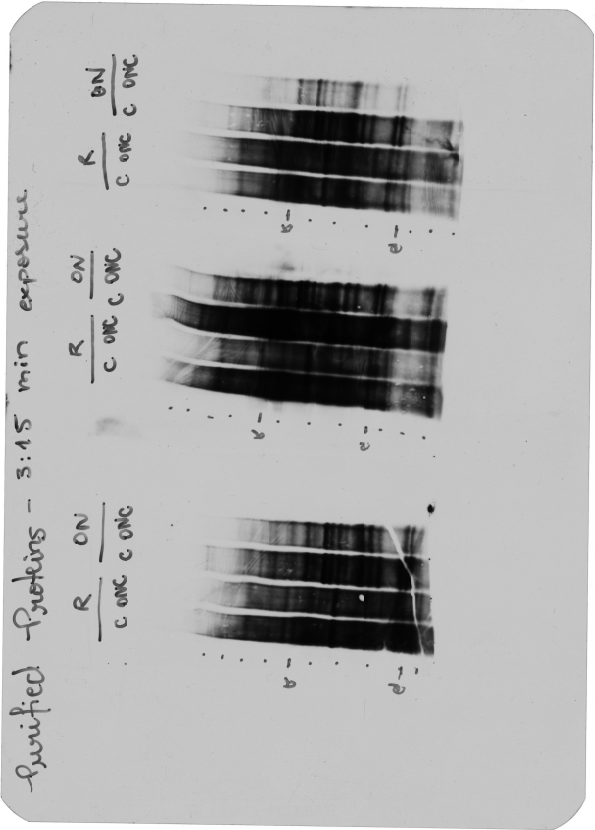

Labelled Lanes

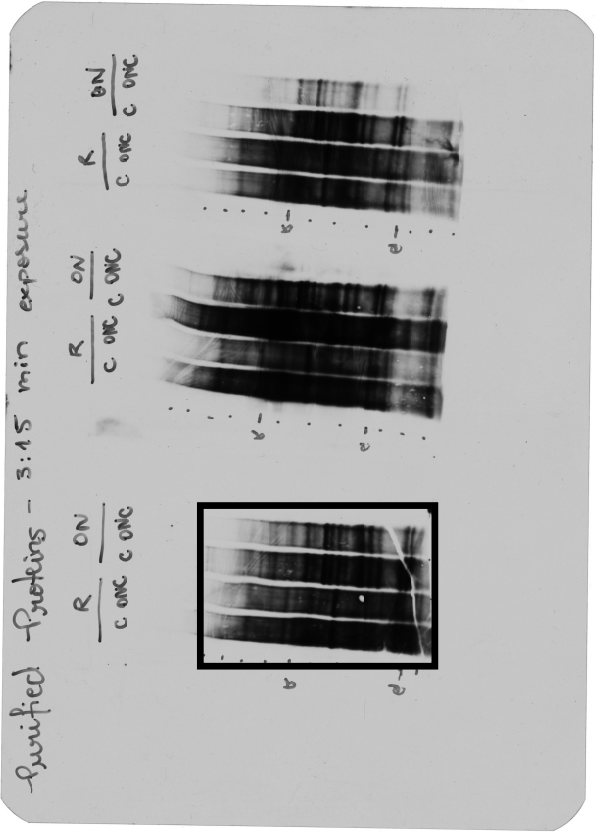

Supplement: Figure 1—source data 2. [file elife-68148-fig1-data2.zip › Figure 1- Source Data 2.pdf]

VHS-A210E  
+  
BIG P N  
ACTH/NF

[2]  
[2.3]  
[2.3]  
[2.3]

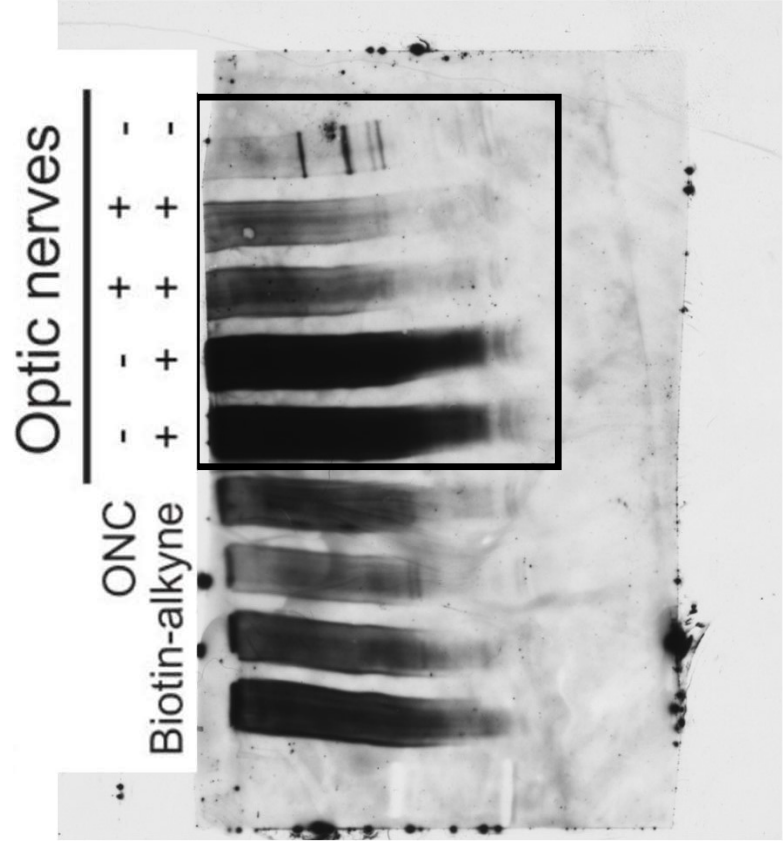

Figure S1b Biotin-beta-alanine

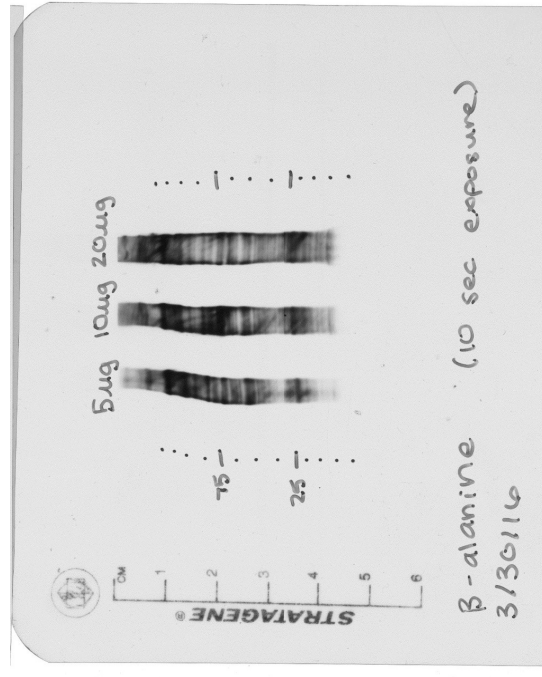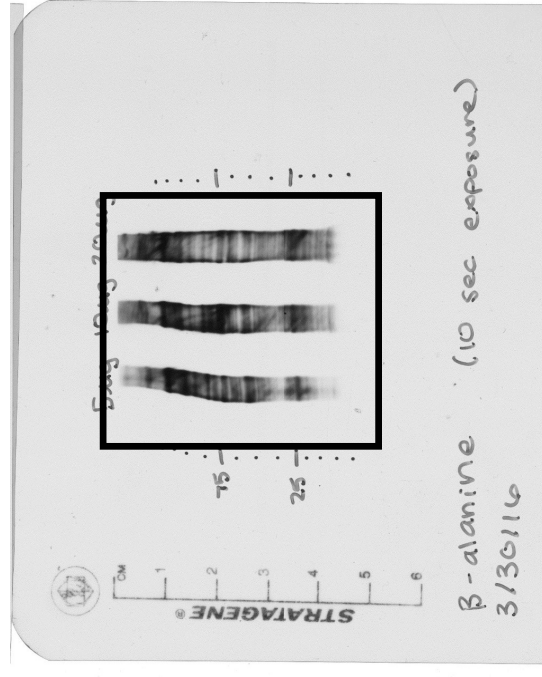

Supplement: Figure 1—figure supplement 1—source data 1. [file elife-68148-fig1-figsupp1-data1.zip › Figure S1- Source Data 1.pdf]

Figure S3 Kif5a<sup>fl/fl</sup> genotyping

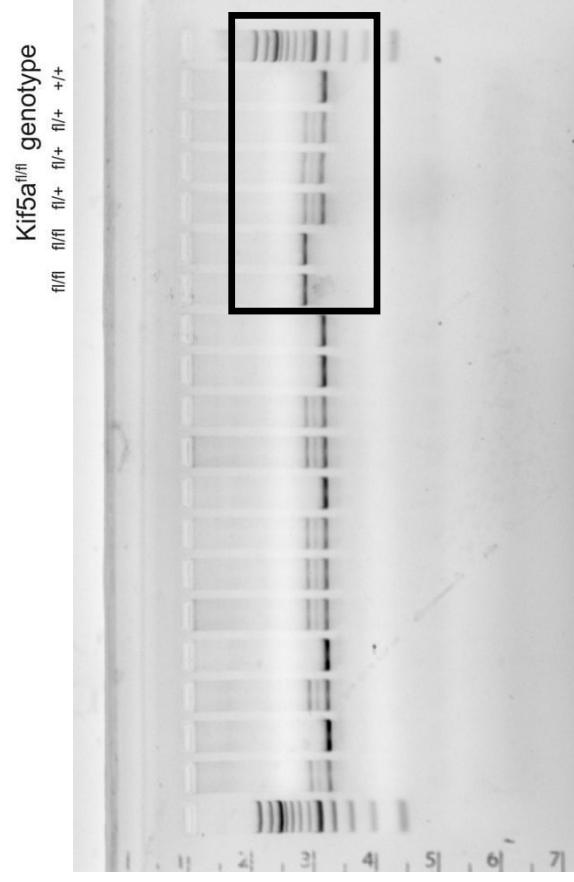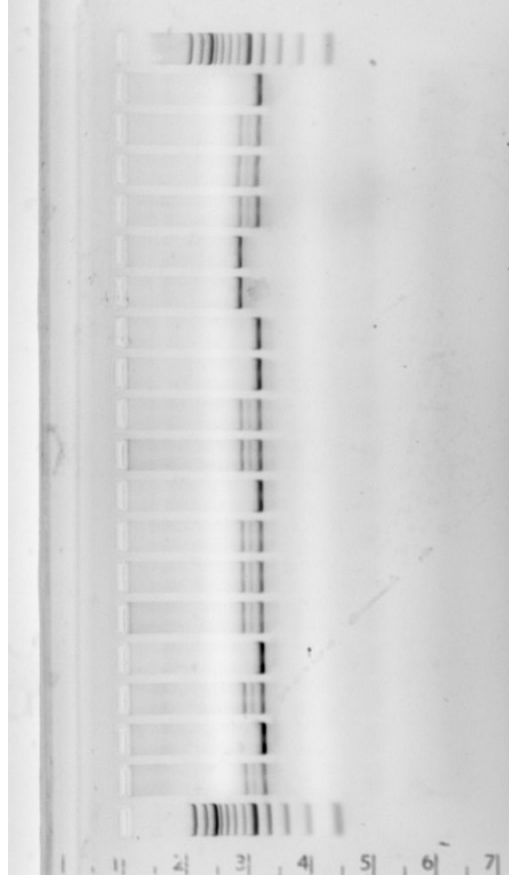

Supplement: Figure 4—figure supplement 1—source data 1. [file elife-68148-fig4-figsupp1-data1.zip › Figure S3- Source Data 1.pdf]

Figure 7a B3Tubulin Immunoprecipitation

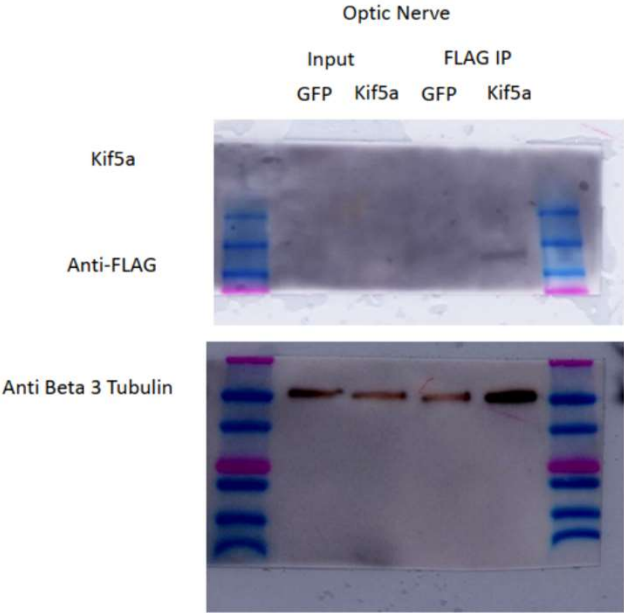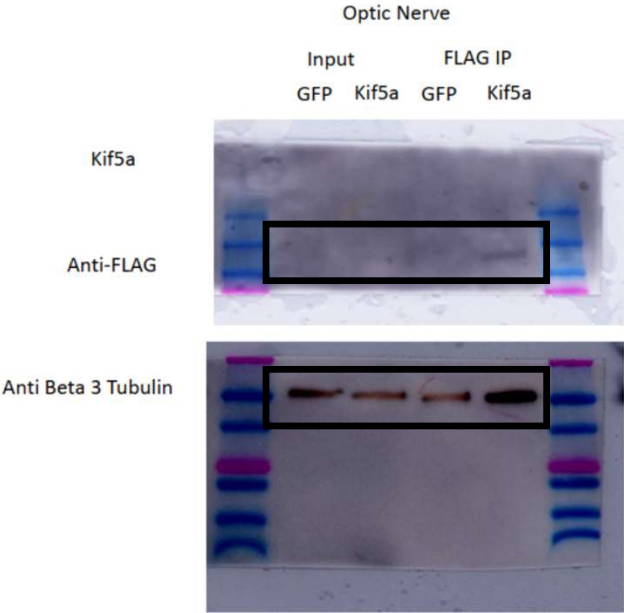

Supplement: Figure 7—source data 1. [file elife-68148-fig7-data1.zip › Figure 7- Source Data 1.pdf]
