# Supplementary material for: Quantitative transportomics identifies Kif5a as a major regulator of neurodegeneration
Source: eLife. 2022 Mar 8;11:e68148. doi: 10.7554/eLife.68148 (PMC8947766; doi:10.7554/eLife.68148)

Figure 2a Sncb western blots

Sncb Inputs

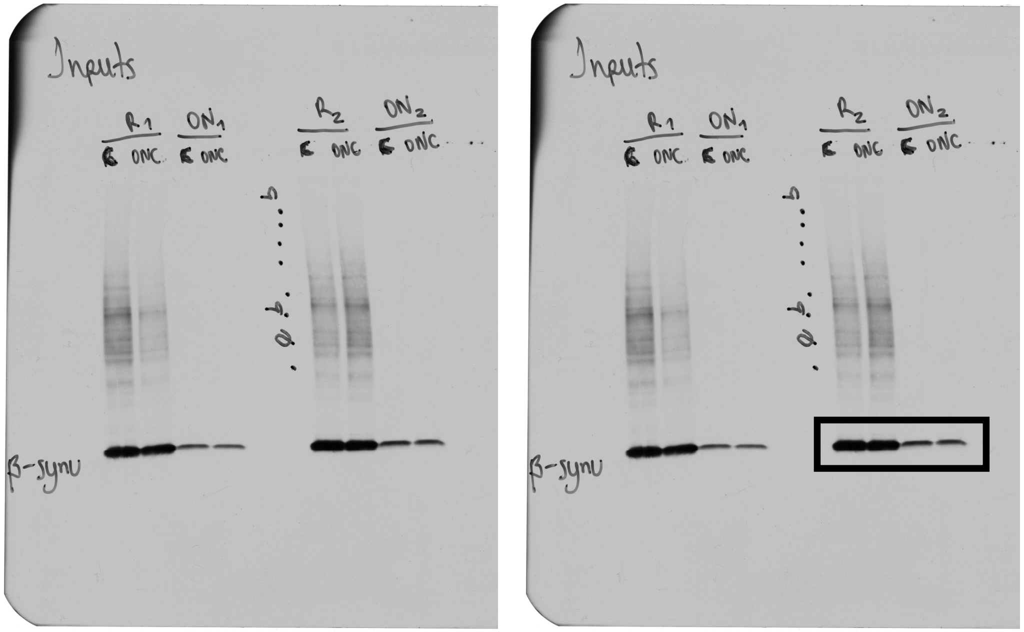

Sncb Immunoprecipitation

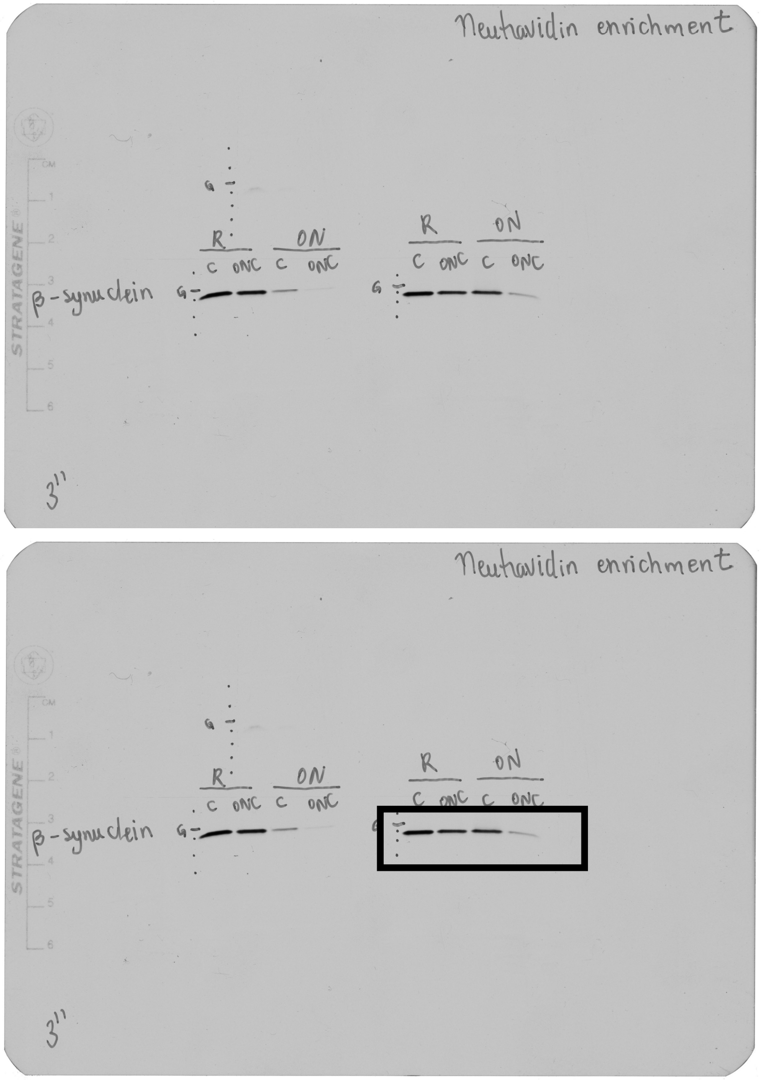

Figure 2a Gap43 western blots

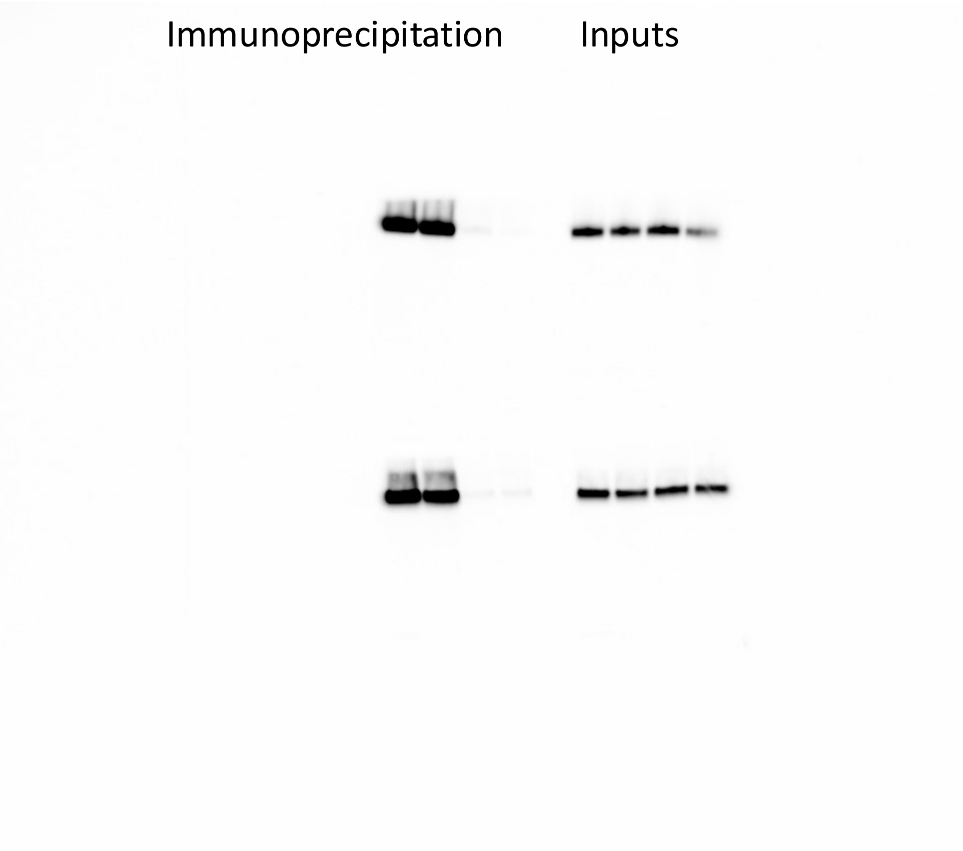

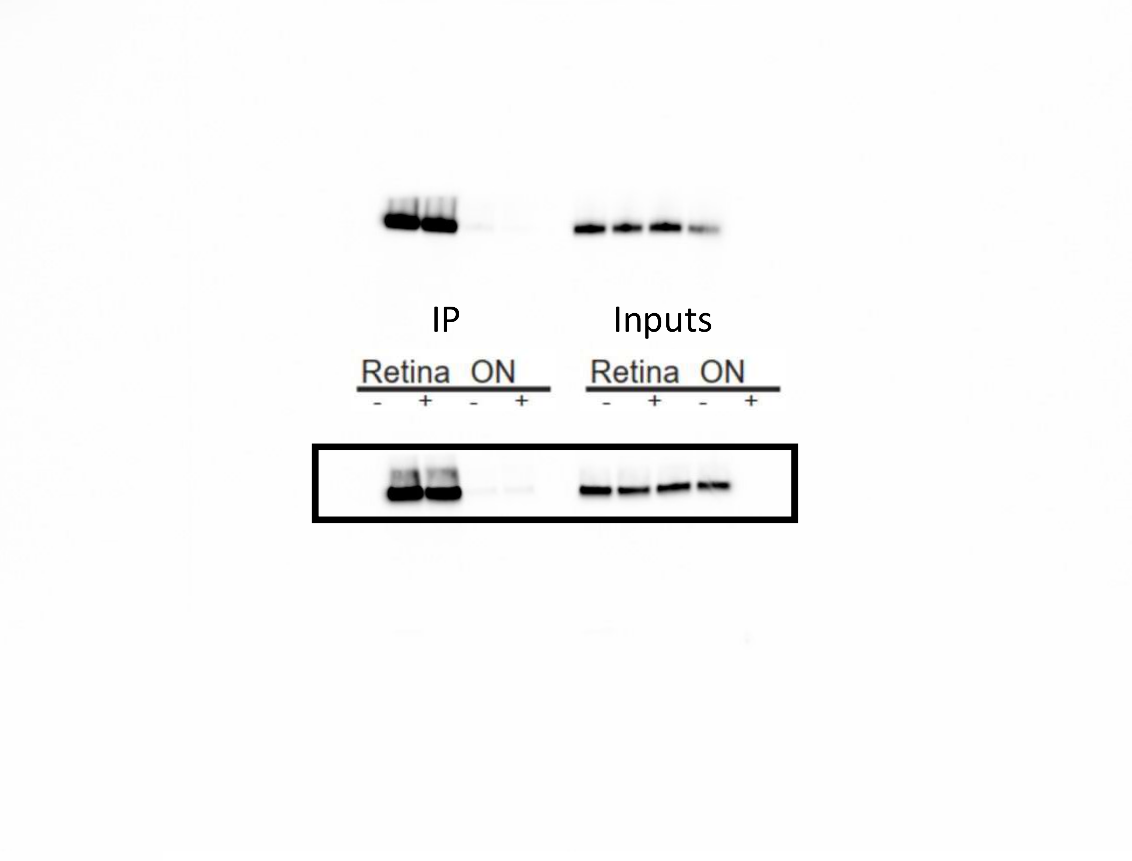

Figure 2a Arf3 western blots

Arf3 Inputs

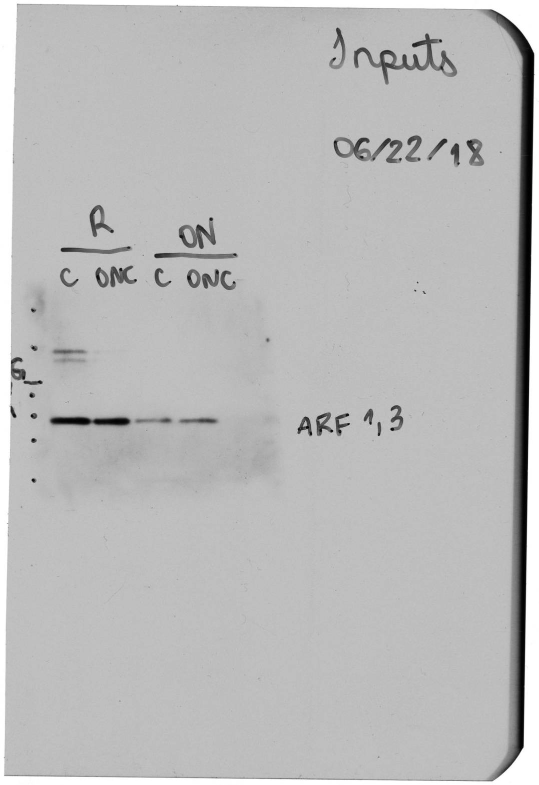

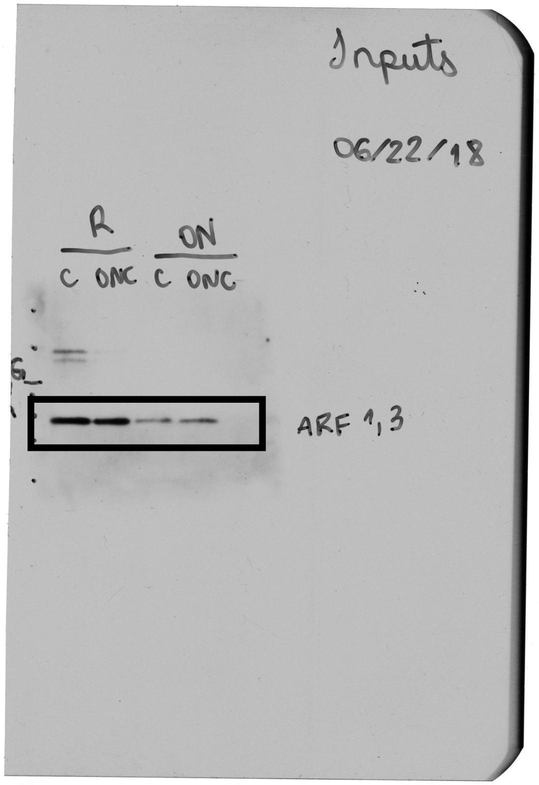

Arf3 Immunoprecipitation

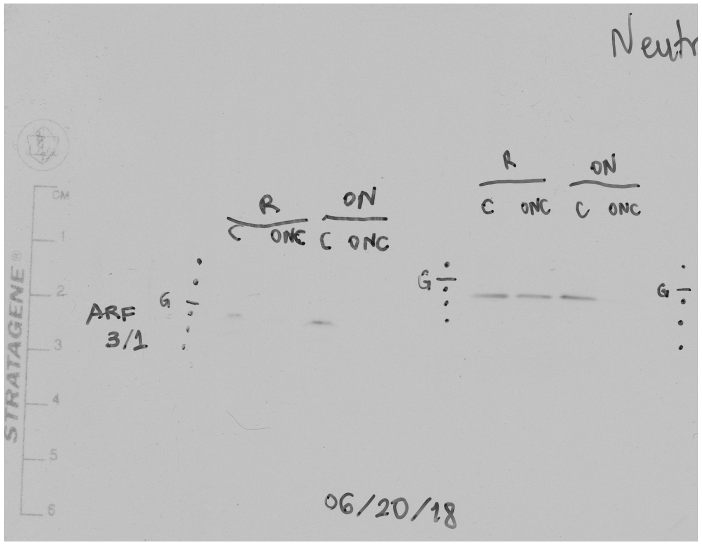

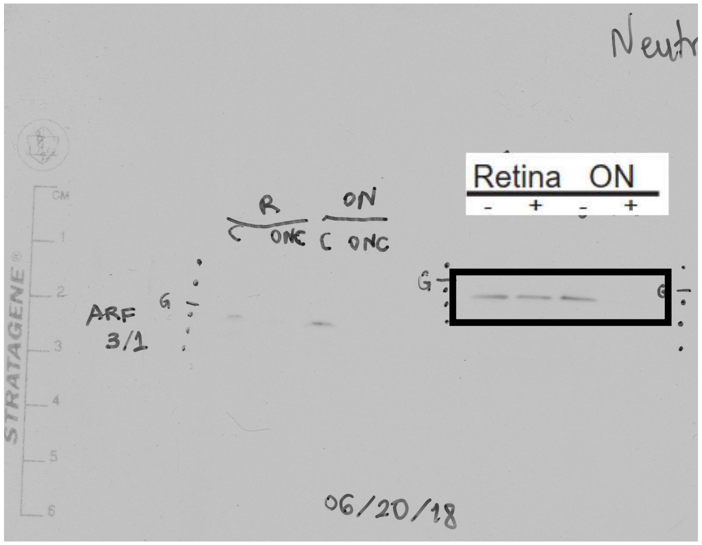

Figure 2d Kif5a WB replicate 1

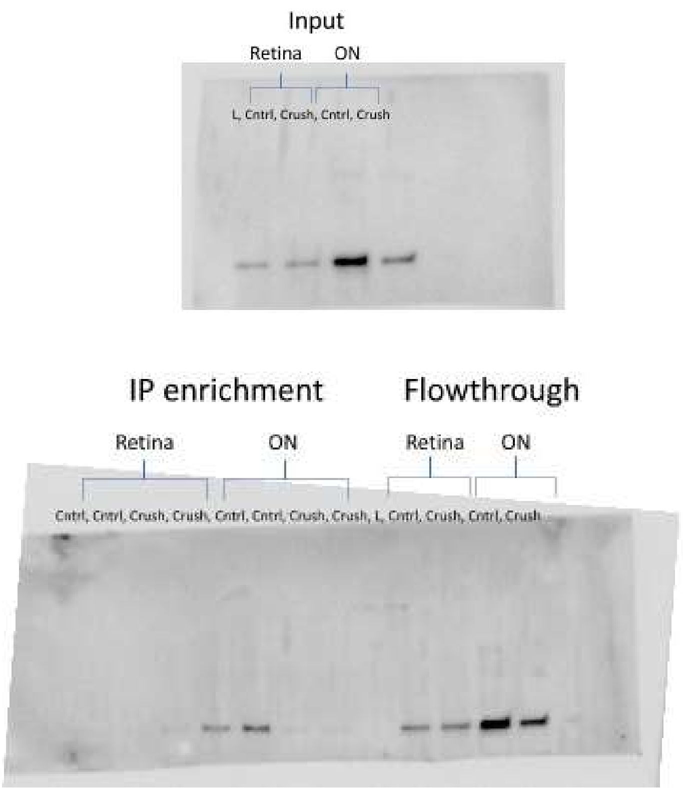

Kif5a

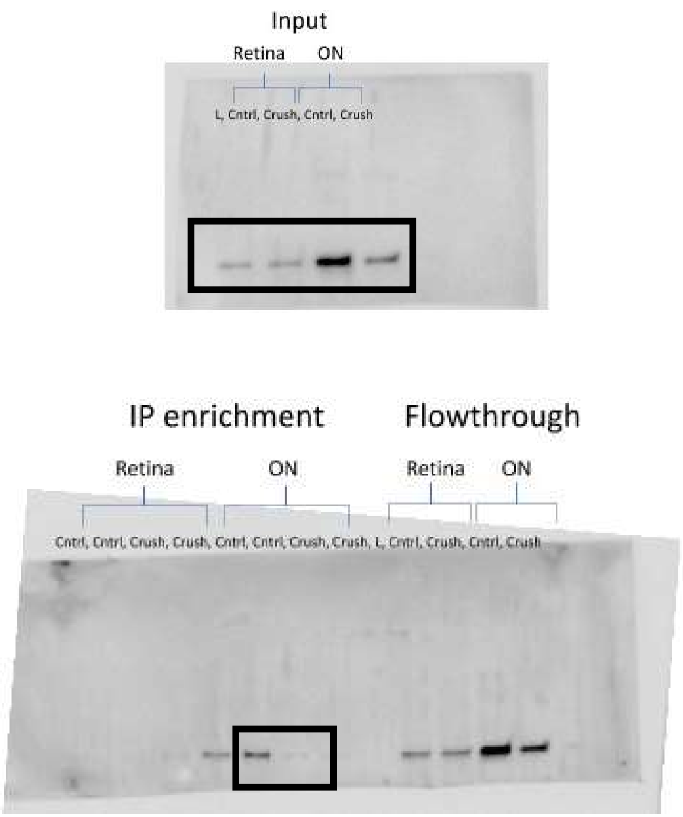

Figure 2d Kif5a WB replicate 2

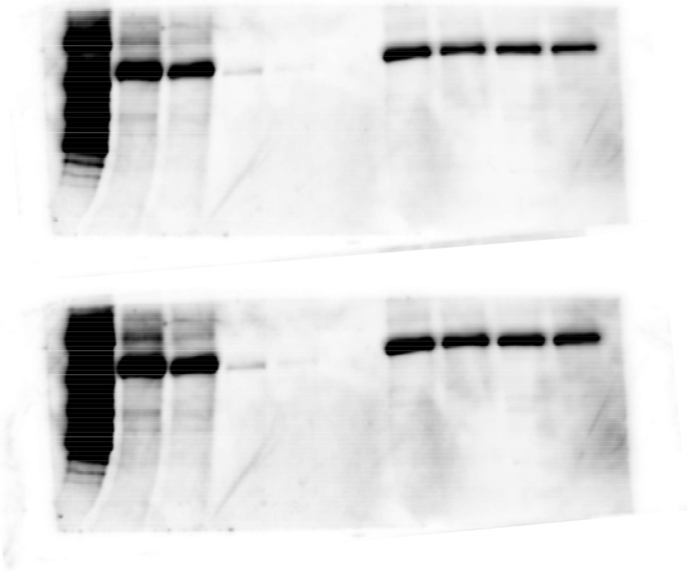

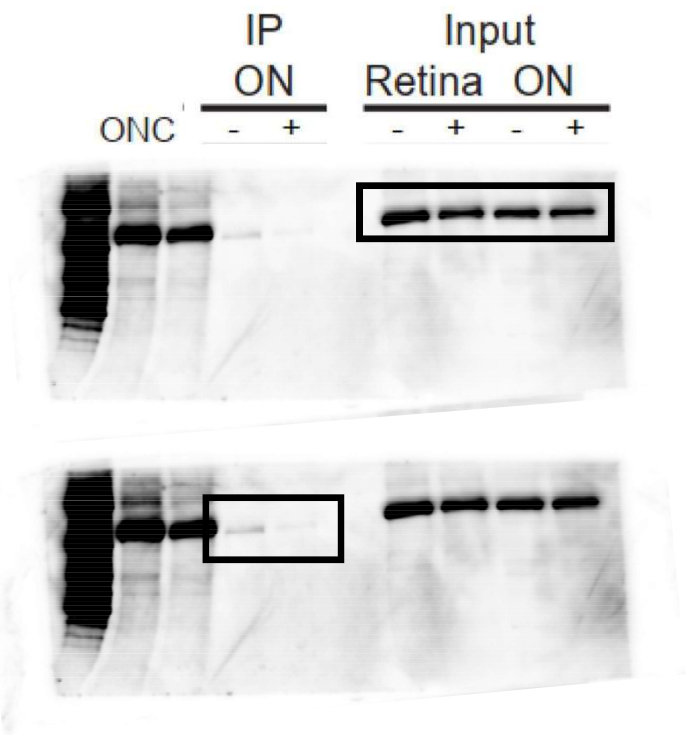

Figure 2d Kif5a WB replicate 3

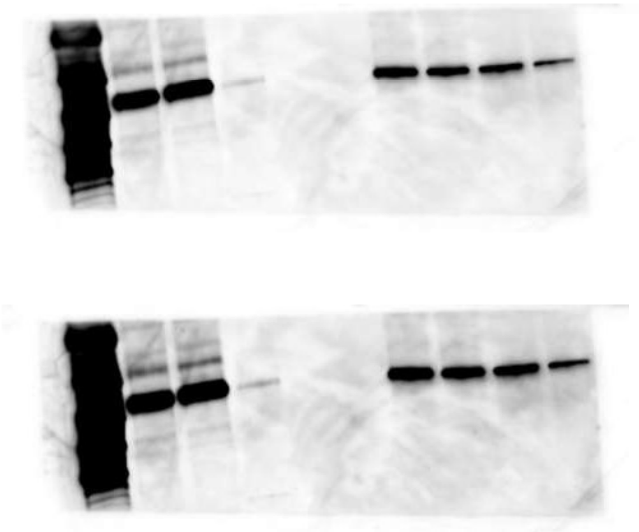

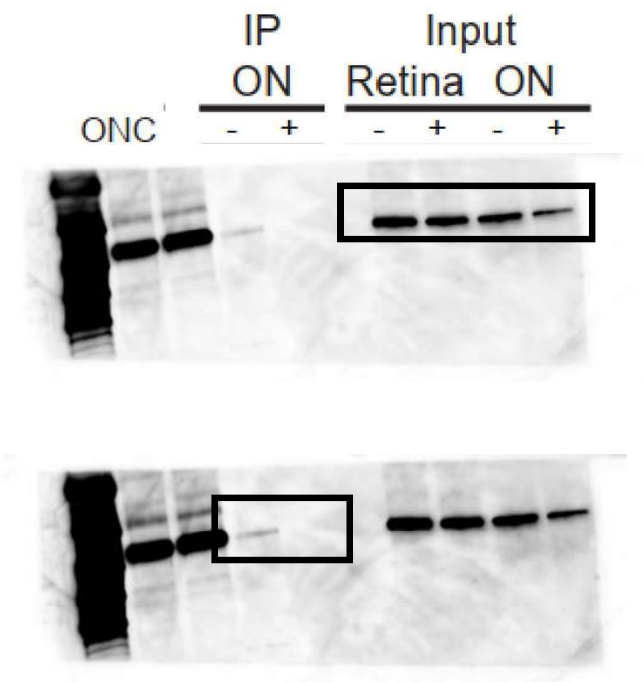

Supplement: Figure 2—source data 1. [file elife-68148-fig2-data1.zip › Figure 2- Source Data 1.pdf]
