# Supplementary material for: Quantitative transportomics identifies Kif5a as a major regulator of neurodegeneration
Source: eLife. 2022 Mar 8;11:e68148. doi: 10.7554/eLife.68148 (PMC8947766; doi:10.7554/eLife.68148)

Figure 4a Kif5a/Kif5c/GAPDH WB

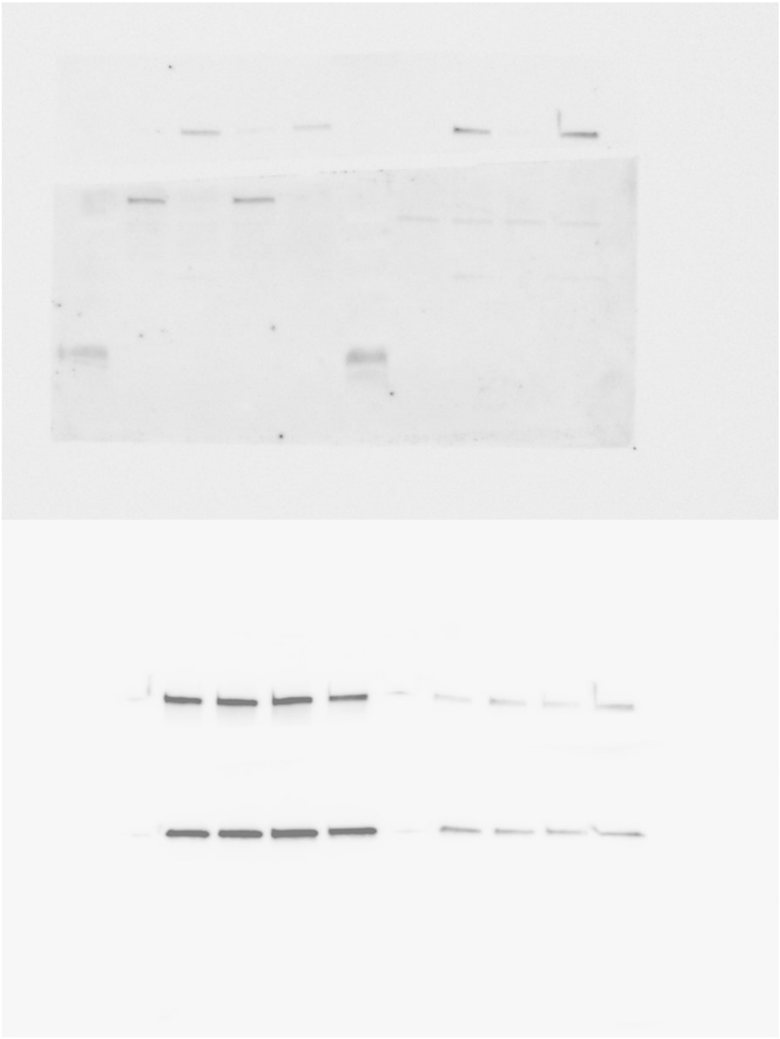

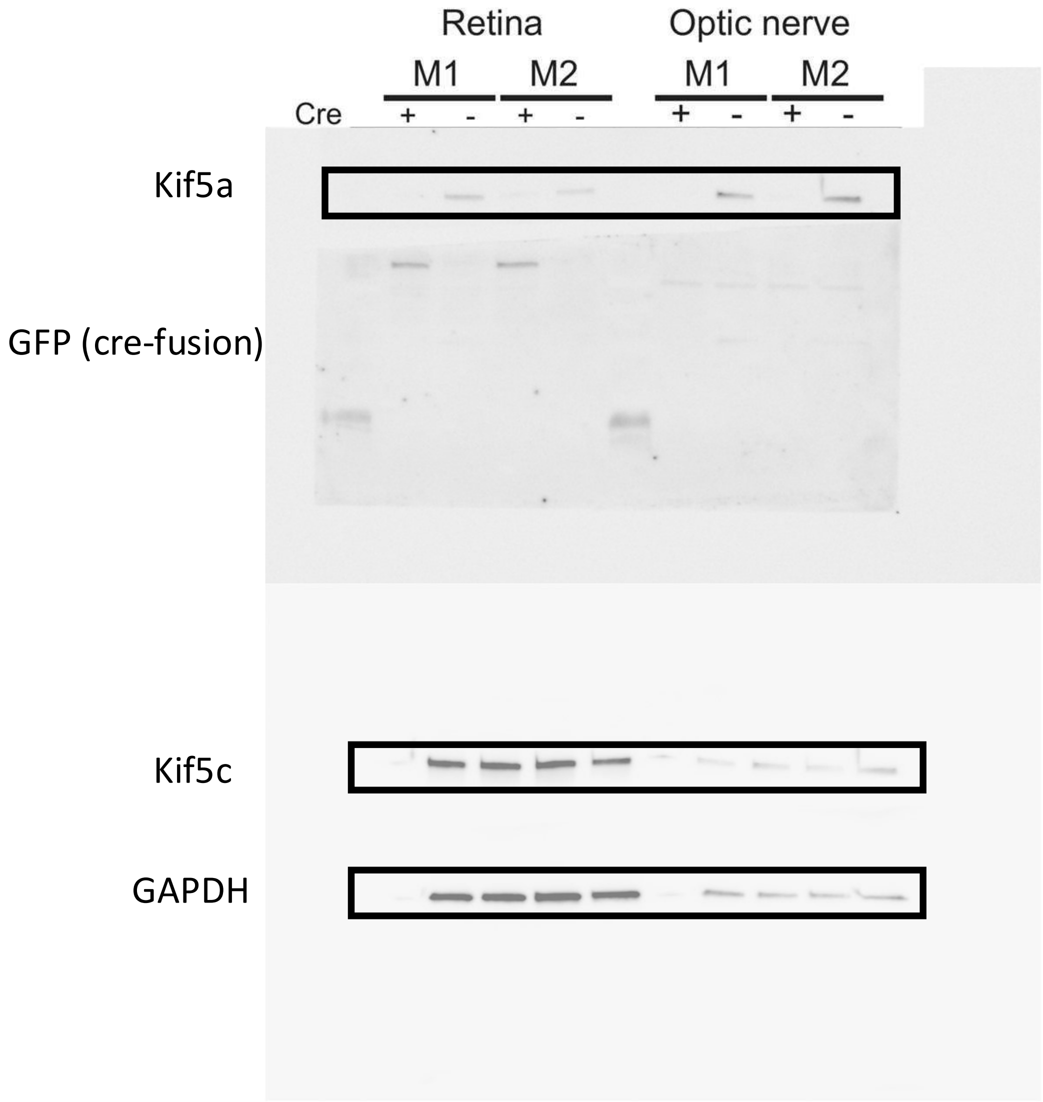

Supplement: Figure 4—source data 2. [file elife-68148-fig4-data2.zip › Figure 4- Source Data 2.pdf]
